# A MALDI-TOF mass spectrometry-based haemoglobin chain quantification method for rapid screen of thalassaemia

**DOI:** 10.1080/07853890.2022.2028002

**Published:** 2022-01-31

**Authors:** Jian Zhang, Zhizhong Liu, Ribing Chen, Qingwei Ma, Qian Lyu, Shuhui Fu, Yufei He, Zijie Xiao, Zhi Luo, Jianming Luo, Xingyu Wang, Xiangyi Liu, Peng An, Wei Sun

**Affiliations:** a State Key Laboratory of Proteomics, Beijing Proteome Research Center, National Center for Protein Sciences (Beijing), Beijing Institute of Lifeomics, Beijing, China; b Beijing Bo'ai Hospital, China Rehabilitation Research Center, Beijing, China; c Longgang District People's Hospital of Shenzhen, Shenzhen, China; d Bioyong Technologics Inc., Beijing, China; e Department of Pediatrics, The First Affiliated Hospital, Guangxi Medical University, Nanning, China; f Beijing Hypertension League Institute, Beijing, China; g Department of Laboratory Medicine, Beijing Tongren Hospital, Capital Medical University, Beijing, China; h Department of Nutrition and Health, China Agricultural University, Beijing, China

**Keywords:** MALDI-TOF, haemoglobin, molecular diagnostics

## Abstract

**Background:**

Thalassaemia is one of the most common inherited monogenic diseases worldwide with a heavy global health burden. Considering its high prevalence in low and middle-income countries, a cheap, accurate and high-throughput screening test of thalassaemia prior to a more expensive confirmatory diagnostic test is urgently needed.

**Methods:**

In this study, we constructed a machine learning model based on MALDI-TOF mass spectrometry quantification of haemoglobin chains in blood, and for the first time, evaluated its diagnostic efficacy in 674 thalassaemia (including both asymptomatic carriers and symptomatic patients) and control samples collected in three hospitals. Parameters related to haemoglobin imbalance (α-globin, β-globin, γ-globin, α/β and α-β) were used for feature selection before classification model construction with 8 machine learning methods in cohort 1 and further model efficiency validation in cohort 2.

**Results:**

The logistic regression model with 5 haemoglobin peak features achieved good classification performance in validation cohort 2 (AUC 0.99, 95% CI 0.98–1, sensitivity 98.7%, specificity 95.5%). Furthermore, the logistic regression model with 6 haemoglobin peak features was also constructed to specifically identify β-thalassaemia (AUC 0.94, 95% CI 0.91–0.97, sensitivity 96.5%, specificity 87.8% in validation cohort 2).

**Conclusions:**

For the first time, we constructed an inexpensive, accurate and high-throughput classification model based on MALDI-TOF mass spectrometry quantification of haemoglobin chains and demonstrated its great potential in rapid screening of thalassaemia in large populations.

**Key messages**
- Thalassaemia is one of the most common inherited monogenic diseases worldwide with a heavy global health burden.
- We constructed a machine learning model based on MALDI-TOF mass spectrometry quantification of haemoglobin chains to screen for thalassaemia.

## Introduction

Thalassaemia is among the most common inherited monogenic diseases worldwide, which is highly prevalent in sub-Saharan African, Mediterranean region, Middle Eastern, Indian subcontinent and Southeast Asian descent [1,2]. About 1–5% of the global population are carriers of genetic thalassaemia mutations [1] and at least 60 000 severely affected individuals were born each year [3]. Thalassaemia causes anaemia, ineffective erythropoiesis, iron overload and other clinical manifestations, which are accompanied by developmental delays and other multiple-organ damages [1,4]. Most patients with severe thalassaemia may die in utero or during early childhood without treatment.

In healthy individuals, haemoglobin contains two α subunits and two β subunits (α_2_β_2_) to work cooperatively to transport oxygen [5]. When α or β subunit encoding gene (*HBA1/HBA2* or *HBB*) has defected, abnormal form or inadequate amount of α or β subunit will cause α- or β-thalassaemia [6–8]. Without intervention, any form of thalassaemia will progress and has increased morbidity with age [9]. Furthermore, the deletion or mutation in *HBA1/HBA2* or *HBB* gene can be inherited by the next generation. So early detection is very important for not only treatment but also the prevention of thalassaemia.

### Thalassaemia diagnosis in clinical practice

In clinical practice, examination of red cell indices and measurement of haemoglobin concentration is used to screen suspected cases of thalassaemia. However, due to the insufficient sensitivity and specificity of these methods, further examinations are still needed. Haemoglobin electrophoresis or high-performance liquid chromatography (HPLC) has been used for the quantification of haemoglobin A2 (Hb A_2_, or α_2_δ_2_) and foetal haemoglobin (Hb F, or α_2_γ_2_). However, the throughput of electrophoresis is limited and HPLC quantification of Hb A_2_ could be interfered with by the existence of Hb Lepore or Hb E variant due to their co-elution with Hb A_2_, which may result in a false increase of Hb A_2_ level [10]. The specificity and sensitivity of these methods for the diagnosis of thalassaemia are not satisfactory. For example, Noppacharn et al. [11] reported that HPLC yielded 76.4% sensitivity and 89.5% specificity for identification α-thalassaemia syndrome in the newborns of Thailand. Genetic analysis (e.g. gap-PCR, DNA sequencing) and family studies are necessary for the final confirmation of thalassaemia [12]. Although next-generation sequencing is more precise than traditional genotyping methods [13], its application is limited by the high cost and additional requirements for bioinformatics analysis.

### Mass spectrometry in thalassaemia

As a kind of high-throughput detection instrument, MALDI-TOF MS (matrix-assisted laser desorption ionization-time of flight mass spectrometry) is playing an increasingly important role in clinical chemistry [14]. Though it has been used for the detection of mutations in thalassaemia patients [15–17], sample pre-treatment (DNA extraction and PCR) is still time-consuming. Direct detection of the intact haemoglobin chains in untreated blood samples is an efficient way to increase the throughput of MALDI-TOF MS. However, there were very few related studies, which only focus on identifying the different peaks of the haemoglobin chain rather than diagnosis. Kleinert and co-workers detected the peaks of wild-type α- and β-globin and the variant β-globin [18]. Iles and Mahmoud revealed the characteristic spectra of thalassaemia [19], then software was developed and the ratio between β-globin and α-globin was used for identification of thalassaemia [20]. However, the diagnostic efficacy of this method was not evaluated. MALDI-TOF MS was only evaluated for newborn sickle cell disease (SCD) screening [21]. Compared with SCD, which is a monogenic disease with a specific haemoglobin variant, thalassaemia is more difficult to be diagnosed due to much more complicated genotypes and phenotypes. Till now, systematic research on the application of MALDI-TOF MS in the diagnosis of thalassaemia in a large population is still lacking. In this study, we constructed a MALDI-TOF MS-based haemoglobin chain quantification method for a rapid screen of thalassaemia, and for the first time, proved the high diagnostic performance of this method in 674 thalassaemia and control samples collected in three hospitals.

## Materials and methods

### Blood sample collection

We recruited 436 individuals who were diagnosed with thalassaemia and 13 non-thalassaemia immediate family members at the First Affiliated Hospital of Guangxi Medical University (Nanning, Guangxi Zhuang Autonomous Region, China) from 2017/05/12 to 2018/1/31. Fasting peripheral blood samples were collected. For those thalassaemia patients who were accepting blood transfusion therapy, samples were collected two weeks after receiving their last blood transfusion. Peripheral blood DNA was extracted for thalassaemia genotyping, which was performed at BGI Clinical Laboratories (Shenzhen, China) using Gap-PCR and SNP detection. Adult haemoglobin 2 (Hb A2, or α2δ2) and foetal haemoglobin (Hb F, or α2γ2) were measured by Bio-Rad Variant II ion-exchange HPLC platforms. Peripheral blood samples from Longgang District People's Hospital of Shenzhen (*N* = 96) and Beijing Bo'ai Hospital (*N* = 129) were collected as control. All the blood samples were collected by EDTA Vacutainer tubes and stored at −80 °C. All individuals provided informed consent and this study was approved by the Ethical Review Boards from the three hospitals above.

### Sample preparation

For each sample, 5 μL of blood was added into 1 mL of dilution buffer (blood peptide mass fingerprinting detection kit 1010306, Bioyong Technologies Inc., Beijing, China) and mixed for 30 s. Then 5 μL of diluted blood solution was added into 5 μL of internal standard protein (myoglobin, 16,952 Da) solution. After adding 10 μL of a sinapinic acid matrix, the solution was mixed and 1 μL of matrix sample mixture was added onto a stainless-steel target plate (S-384-D, Bioyong Technologies Inc., Beijing, China) for mass spectrometry analysis.

### Mass spectrometry analysis and data processing

The mass spectrometric analysis of the samples was carried out after mass calibration on a MALDI-TOF mass spectrometer (Clin-TOF-II; Bioyong Technologies Inc., Beijing, China) in a positive linear mode with an m/z range between 2,000 and 20,000. Each spectrum was accumulated with 500 laser shots (50 positions per sample spot and 10 laser shots per position). The MALDI-TOF raw data were processed with MALDI-MS software (V2.9.3). The *m/z* and peak intensity values of all peaks were extracted as ASCII text files after smooth and baseline removal. Then the files were imported into a self-compiler program BE-D. After *m/z* was calibrate with the internal standard, the peaks corresponding to alpha, beta and gamma haemoglobin were selected with internal standard (*m/z* tolerance error was 3000 ppm) and the relative intensities of these peaks were extracted in batches. The intensities of alpha, beta and gamma haemoglobin peaks in each spectrum were divided by the intensity of the internal standard peak as normalisation.

### Data analysis

Correlation analysis was performed with Spearman rank correlation using R 3.5.3. PCA analysis was performed with factoextra package in R 3.5.3. AUC value was calculated in R 3.5.3 with the pROC package [22]. The classification model of random forest (RF), logistic regression (LR), support vector machine (SVM), K-nearest neighbour (KNN), decision tree (DT), naive Bayes (NB), adaptive boosting (Adaboost) and artificial Neural Network (ANN) were performed using R 3.5.3 with randomForest [23], glmnet [24], e1071 [25], kknn [26], rpart, e1071, adabag [27] and nnet [28] package, respectively.

## Results

### MALDI-TOF detection of blood samples

**Figure 1.**
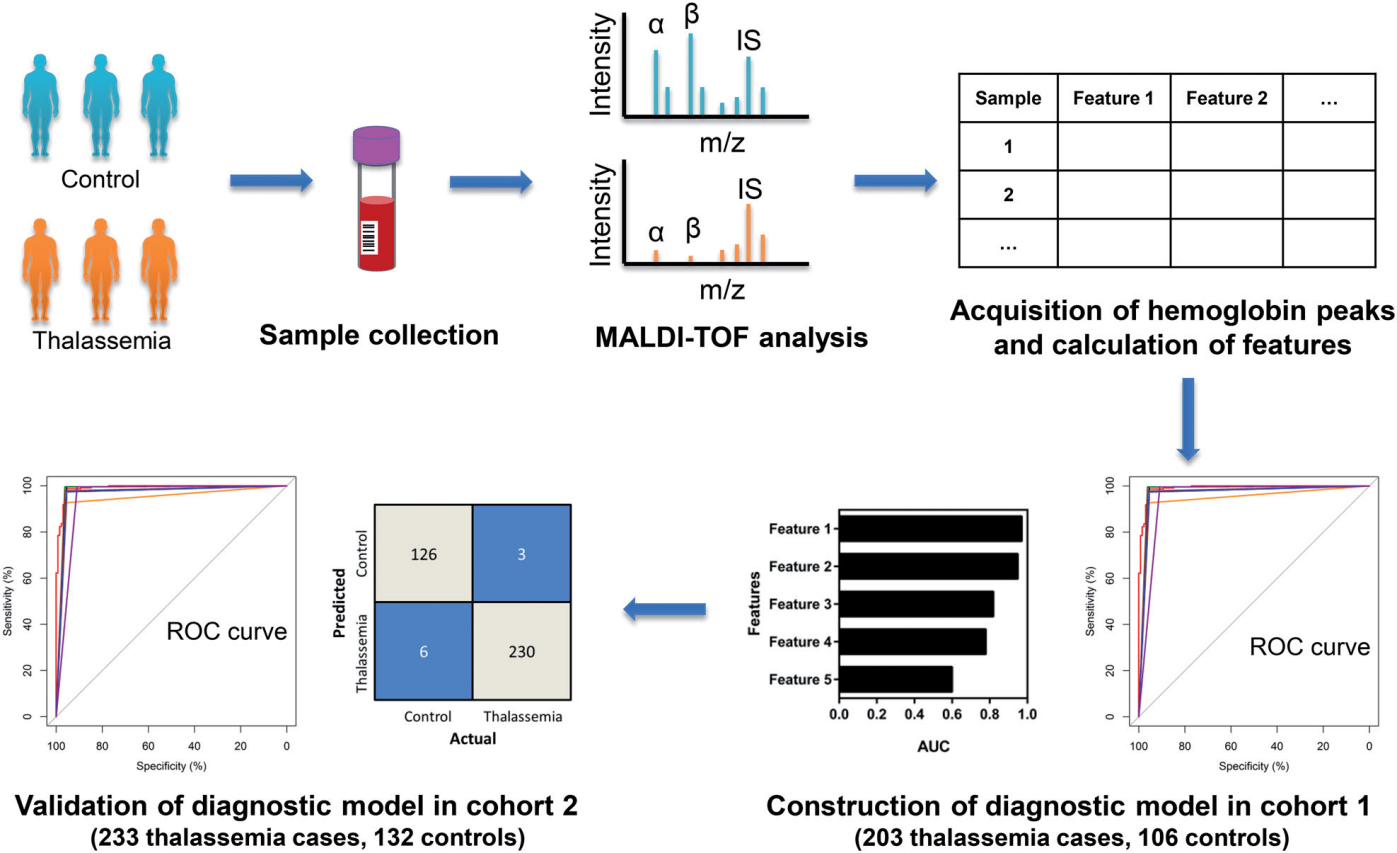
Scheme of establishing a diagnostic model for rapid screening of thalassaemia patients. The serum samples collected from thalassaemia patients and control participants were analysed with MALDI-TOF after simple pre-treatment. The α-globin, β-globin, γ-globin and internal standard (IS) peaks were selected, and corresponding features were used to establish the diagnostic models with different machine learning methods in cohort 1. Then the diagnostic models were verified in cohort 2.

Peripheral venous blood samples from 436 diagnosed thalassaemia patients with genotyping results and 238 control individuals were enrolled in this study. The clinical information of these participants was shown in Table S1. The samples were analysed by MALDI-TOF MS in two batches. Samples analysed in the first batch were assigned to cohort 1, including 203 thalassaemia samples and 10 control samples from First Affiliated Hospital of Guangxi Medical University (Hospital A) and 96 control samples from Longgang District People's Hospital of Shenzhen (Hospital B), while samples analysed in the second batch were assigned to cohort 2, including 233 thalassaemia samples and 3 control samples from Hospital A and 129 control samples from Beijing Bo'ai Hospital (Hospital C). After peak extraction and alignment, the relative intensities of haemoglobin related feature peaks in cohort 1 were used for diagnostic model construction, while those in cohort 2 were used for diagnostic model validation (shown in Figure 1).

The peak intensities of α-globin, β-globin, γ-globin and internal standard were obtained with both 1+ and 2+ charged ions. The representative mass spectra of haemoglobin and internal standard peaks in samples from different groups were shown in Figure S1. In order to improve quantitative accuracy, the intensities of these haemoglobin peaks were normalised by the internal standard peak. The ratio between α-globin and β-globin (α/β) was calculated as a feature to detect thalassaemia according to the research by Iles and Mahmoud [19]. In addition, α-globin subtracting β-globin (α-β), which showed a significant difference between the thalassaemia group and the control group (Figure S2), was also used as a feature. In total, 10 features were used in this study for further feature selection and classification model construction.

### Characteristics of haemoglobin related feature peaks

**Figure 2.**
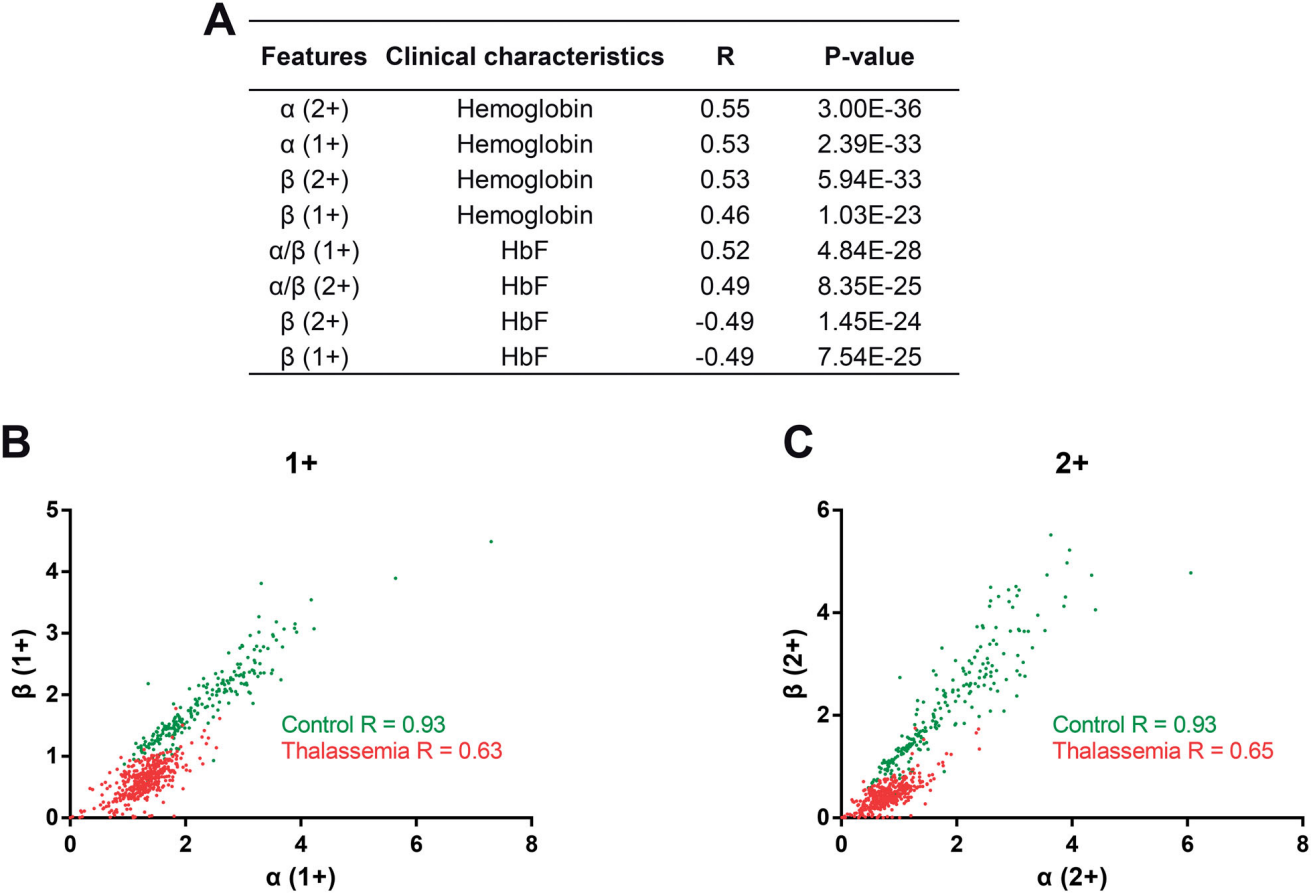
Correlation analysis of haemoglobin related features in thalassaemia patients. (A) The spearman correlation analysis between haemoglobin related features and clinical characteristics in thalassaemia patients. (B) The scatter plot showed that the correlation between α-globin and β-globin (both 1+ charged) is different between thalassaemia group and the control group. (C) The scatter plot showed that the correlation between α-globin and β-globin (both 2+ charged) is different between thalassaemia group and the control group. Outliers in the scatter plot are not shown for clarity.

The relationship between 10 features from MALDI-TOF and clinical characteristics of thalassaemia patients was also analysed. We found that the level of α-globin or β-globin (both 1+ and 2+ charged) was positively correlated with the blood concentration of haemoglobin (Figure 2A). While the level of β-globin (both 1+ and 2+ charged) was negatively correlated with Hb F, and the ratio between α-globin and β-globin (α/β, both 1+ and 2+ charged) was positively correlated with Hb F. These results indicate that the features based on MALDI-TOF can effectively reflect the haemoglobin-related changes in thalassaemia patients. In addition, we found that the correlation between α-globin and β-globin levels in the control group is higher than that in the thalassaemia group for both charged ions (Figure 2B and Figure 2C), indicating the imbalanced level of α-globin and/or β-globin in thalassaemia patients, which will be the underlying mechanism basis for diagnostic model construction.

### Diagnostic model construction in cohort 1

**Figure 3.**
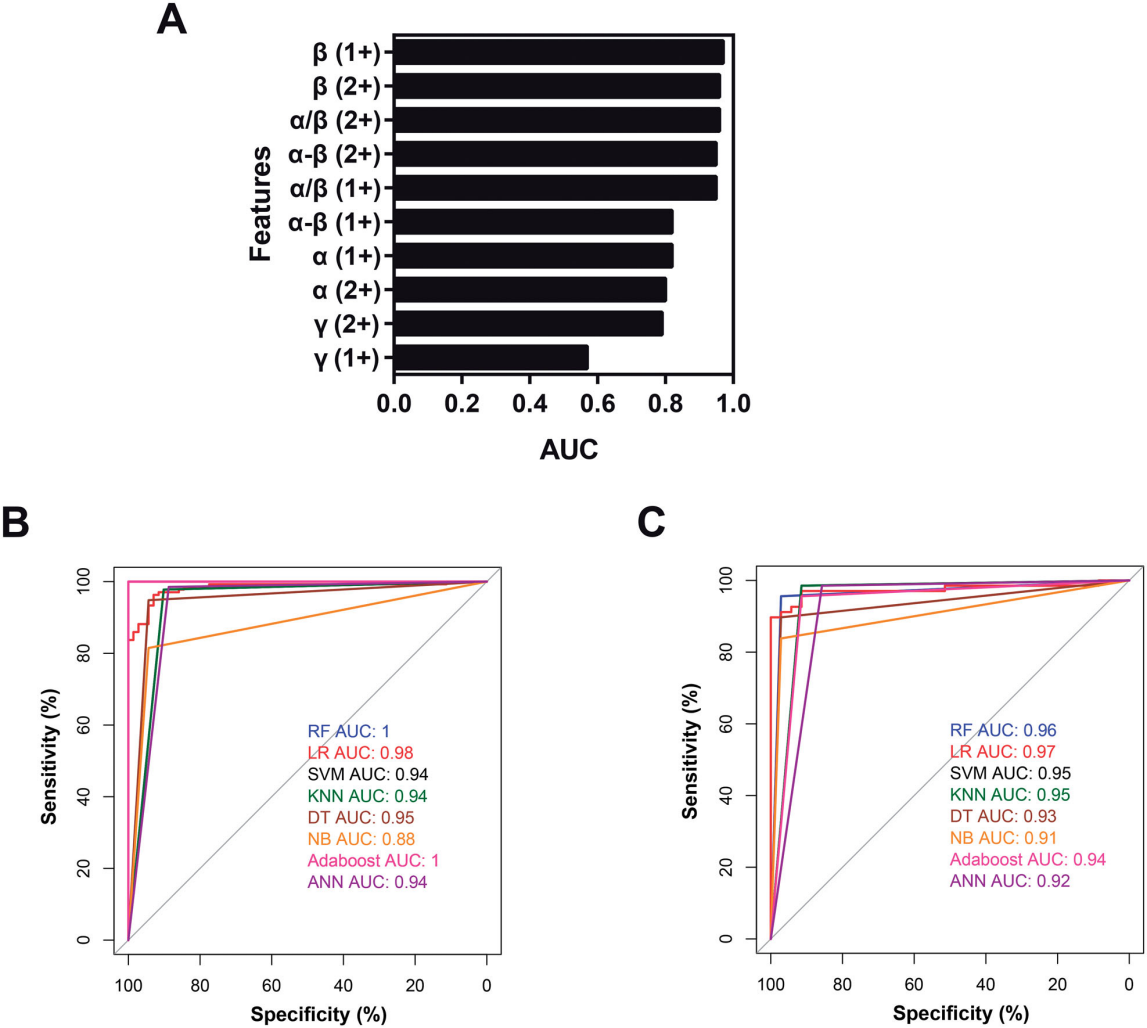
Diagnostic model construction in cohort 1. (A) The AUC value of each feature for distinguishing the thalassaemia patients from controls in cohort 1. (B) ROC curves of eight different machine learning models in the training dataset. (C) ROC curves of eight different machine learning models in the test dataset.

The PCA analysis based on these 10 features demonstrated that the thalassaemia patients could be clearly separated from control individuals (Figure S3). To further select features which could be used in the diagnostic models, the area under the curve (AUC) of the receiver operating characteristic (ROC) curves for each feature in cohort 1 was calculated to evaluate their performance for diagnosis of thalassaemia (Figure 3A). Five features got AUC above 0.95, including 1+ charged β-globin, 2+ charged β-globin, 2+ charged α/β, 2+ charged α-β, and 1+ charged α/β. Among them, the AUC of 1+ charged β-globin reached 0.97 (95% confidence interval [CI] 0.96–0.99). In order to get desirable distinguishing efficiency, these top 5 features were further used for classification model construction by machine learning methods. First, the samples in cohort 1 were randomly split into training and test datasets with an allocation of 2:1, corresponding to 206 (135 patients and 71 controls) and 103 (68 patients and 35 controls) samples, respectively. Totally, eight machine learning methods including random forest (RF), logistic regression (LR), support vector machine (SVM), K-nearest neighbour (KNN), decision tree (DT), naive Bayes (NB), adaptive boosting (Adaboost) and artificial Neural Network (ANN) were used to construct classification models in the training dataset. As shown in Figure 3B, all models except NB got AUC values greater than 0.90. The AUC values of RF, LR and Adaboost were above 0.97. Then the classification efficiency of these models was validated in the test dataset. All of the eight models obtained in the training dataset successfully distinguished thalassaemia patients from controls in the test dataset with AUC above 0.9 (Figure 3C). And LR model got the best classification performance with an AUC of 0.97 (95% CI 0.94–1) in the test dataset.

### Validation of diagnostic model efficiency in cohort 2

**Figure 4.**
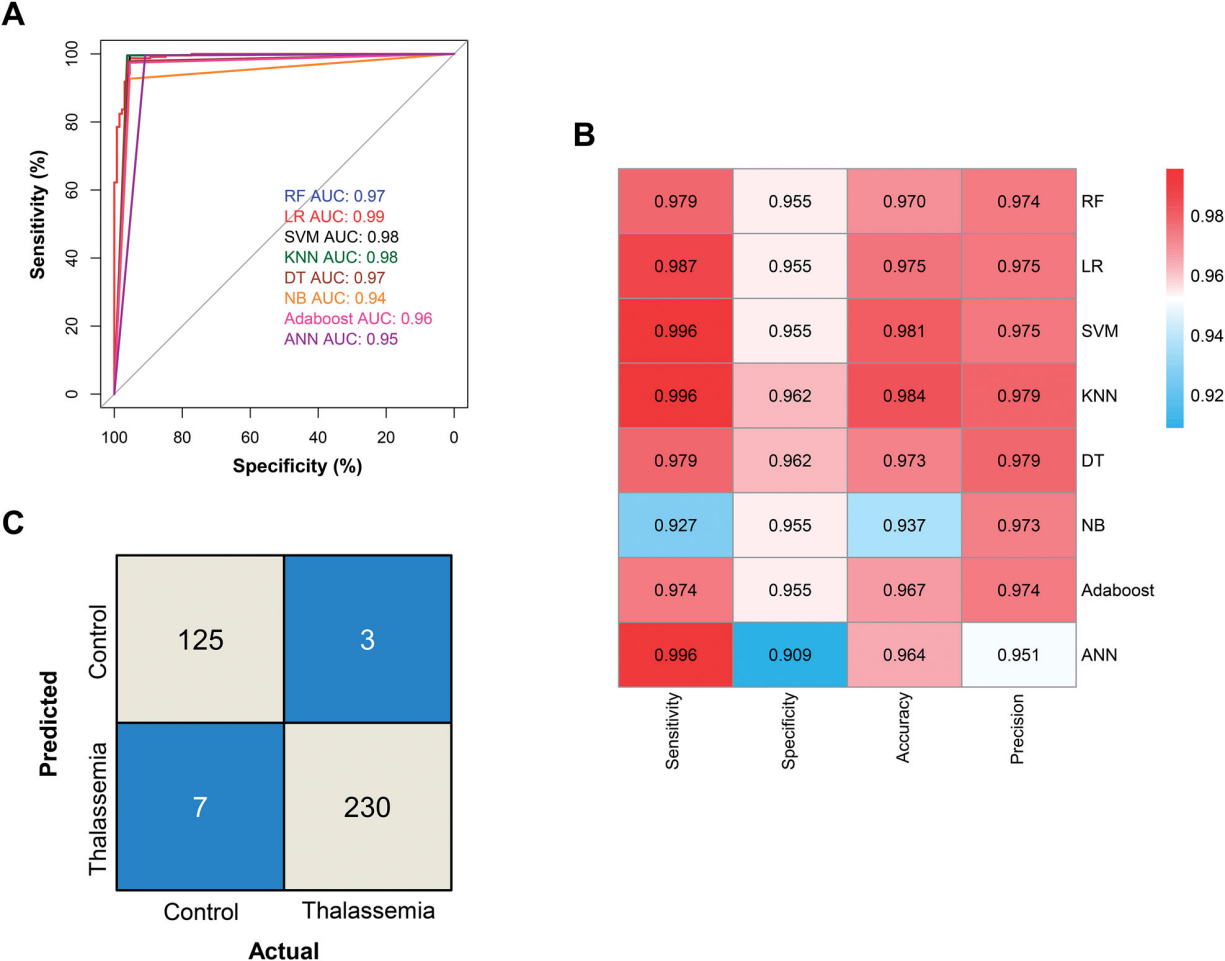
Thalassaemia diagnostic model validation in cohort 2. (A) ROC curves for eight ML algorithms. (B) Summary of the sensitivities, specificities, accuracies and precisions obtained for each ML algorithm model. (C) The confusion matrix of the classification results by the LR model.

Finally, we tested the classification efficiency of these 8 models in an independent validation cohort 2, which consists of 233 thalassaemia patients and 132 controls. All the eight models could distinguish thalassaemia patients from controls with AUC greater than 0.94 (Figure 4A), among them LR model got the best AUC of 0.99 (95% CI 0.98–1). The sensitivity, specificity, accuracy and precision of the eight models were shown in Figure 4B. The sensitivity of our method is significantly higher than that of HPLC in samples detected in this study (47.4%, based on HbA2 with a 3.5% cutoff). Interestingly, the sensitivity obtained by all models except NB is higher than the specificity, indicating that the classification models based on MALDI-TOF MS are more suitable for screening thalassaemia patients in the population. The classification accuracy of most models exceeded 0.96, while the classification precision of most models reached 0.97. Since the LR model achieved good classification performance in the training and test datasets in cohort 1, and independent validation cohort 2, it is recommended for future applications in the screening of thalassaemia. The confusion matrix of the LR model in the independent validation cohort 2 is shown in Figure 4C. Among 233 thalassaemia patients and 132 control cases in validation cohort 2, only 3 thalassaemia patients and 7 control individuals were misclassified (all of the misclassified samples by LR model in two cohorts were shown in Table S2). This result in the independent validation cohort demonstrated that the MALDI-TOF-based classification model can effectively distinguish thalassaemia patients from control cases and has a great potential for thalassaemia screening in the population.

### Identification of α- and β-thalassaemia

In addition, we found that the classification model based on MALDI-TOF can also be used to identify β thalassaemia. Based on the thalassaemia genotyping results in Hospital A (Table S1), we divided the samples into β-thalassaemia group (including β-thalassaemia and αβ compound thalassaemia samples) and non-β-thalassaemia group (including α-thalassaemia samples and healthy controls). This resulted in 151 β-thalassaemia samples and 157 non-β-thalassaemia samples in cohort 1, and 201 β-thalassaemia samples and 164 non-β-thalassaemia samples in cohort 2. Six features with AUC > 0.85 in cohort 1 were selected to construct the classification model (Figure S4), including 1+ charged β-globin, 2+ charged β-globin, 1+ charged α/β, 2+ charged α/β, 1+ charged α-β, and 2+ charged α-β. Then the samples in cohort 1 were randomly split into training and test datasets with an allocation of 2:1. The classification efficiency of eight machine learning models was calculated in the training and test datasets in cohort 1 and the validation cohort 2, sequentially. With the best and most stable classification performance (Table S3), the LR model is recommended for the identification of β-thalassaemia (AUC = 0.94, 95%CI 0.91–0.97, sensitivity = 96.5%, specificity = 87.8% in the validation cohort). However, MALDI-TOF based classification model cannot effectively identify α-thalassaemia with AUC ≤ 0.72 in the validation cohort.

## Discussion

Considering its high prevalence in low and middle-income countries, an inexpensive, accurate and high-throughput screening test of thalassaemia prior to a more expensive confirmatory diagnostic test is urgently needed. In this study, we constructed a MALDI-TOF MS-based haemoglobin chain quantification method and a corresponding MS data algorithm model for rapid screening of thalassaemia. The LR model with 5 haemoglobin peak features achieved good classification performance both in cohort 1 and independent validation cohort 2 (AUC 0.99, sensitivity 98.7%, specificity 95.5%). Based on the analysis of 674 thalassaemia and control samples collected in three hospitals, this study became the first application of MALDI-TOF MS in the diagnosis of thalassaemia in a large population.

Despite of the high performance of this screening method, there were still 3 thalassaemia patients and 6 control individuals misclassified in cohort 2. The misclassification of control individuals might be partly due to the limitation of genotyping, by which the rare thalassaemia mutations could not be detected by traditional methods [13,29]. For example, when we review the medical history retrospectively, we found that case A0028, which was assigned to the control group based on genotyping results, has a history of thalassaemia therapy. That means this case may be a thalassaemia patient with a rare mutation not included by genotyping assays. Considering this advantage over traditional genotyping, the actual specificity of our method may be higher. On the other hand, the miss-diagnosed thalassaemia patients by MALDI-TOF MS were all α-thalassaemia cases, meaning that β-thalassaemia patients were all successfully detected. This result suggested that our MALDI-TOF MS method is more sensitive for the detection of β-thalassaemia, which is usually more serious than α-thalassaemia, considering mutations in more (at least three) copies of *HBA* are required in symptomatic α-thalassaemia patients [30].

What’s more, our study showed that MALDI-TOF MS can also be used to specifically identify β-thalassaemia. LR classification model with 6 haemoglobin peak features demonstrated an AUC of 0.94, the sensitivity of 96.5% and specificity of 87.8% for identification of β-thalassaemia in the validation cohort 2. However, MALDI-TOF MS-based classification model cannot effectively identify α-thalassaemia. The relatively poor performance of the two models for α-thalassaemia detection might be related to the relatively low distinguishing ability of α-globin, which was not in the selected top 5 or 6 features included in the thalassaemia or β-thalassaemia models.

It is worth notice that near 46% (200/436) of thalassaemia patients are accepting blood transfusion therapy. The excellent performance of the model represented the robustness and the anti-interference ability of the MALDI-TOF MS platform. In addition, both asymptomatic carriers and symptomatic patients of thalassaemia can be detected through this method, which will be helpful for not only treatment but also prevention of thalassaemia.

Compared with common MALDI-TOF MS-based methods for disease diagnosis, our thalassaemia screening method has several unique characteristics, which may contribute to its outstanding performance: (1) Theoretically, the application of MALDI-TOF MS in thalassaemia screening is based on the quantification of globin subunits (e.g. α and β), which are the molecular pathogenic basis for thalassaemia. The features we selected for model construction, including α-globin, β-globin, and α/β were correlated with haemoglobin indices (Figure 2A) and reflected the imbalanced level of α-globin and/or β-globin in thalassaemia (Figure S2, Figure 2B and Figure 2C). (2) Technically, an internal standard was used in mass spectrometry for normalisation to improve the quantitative reproducibility and accuracy [31]. In this study, myoglobin, whose molecular weight is close to those of haemoglobin chains was used as an internal standard, so that all the feature peaks can be clearly distinguished by MALDI-TOF MS. 3) Thirdly, comprehensive evaluation of both 1+ and 2+ charged peak intensities with 8 machine learning methods was performed for optimisation of the effect of the model.

Compared with genotyping or HPLC, the much less time and cost used by MALDI-TOF MS make it more suitable for rapid screening of thalassaemia in high-risk populations. Without any sample pre-treatment process or expensive consumables, it takes only about 1 min and less than 1 US dollar to analyse one sample. Only 5 μL of untreated blood is needed for each person, which makes it feasible for analysis of heel blood in newborns or fingerstick blood in adults. However, MALDI-TOF MS-based thalassaemia screening cannot predict the exact thalassaemia genotype according to globin peaks. Thus, a further genetic test is still needed to guide clinical treatment. Another shortcoming in this study is that individuals younger than 3 years were not included. Considering that the levels of haemoglobin subunits are different in newborns, infants and adults, a subsequent study on thalassaemia screening in newborns and infants is needed in the future.

In conclusion, we performed the first MALDI-TOF MS-based thalassaemia screening study with more than 600 cases from the multi-center population, which demonstrated that the MALDI-TOF MS-based classification model can effectively, rapidly, and cheaply detect thalassaemia patients and has a great potential for thalassaemia screening in large populations.

## Supplementary Material

Supplemental Material

## Data Availability

The data of this study are available within the article and supplementary information. Further information about the raw data of MALDI-TOF is available from the corresponding authors, upon reasonable request.

## Abbreviations

HPLC: high-performance liquid chromatography
Hb A2: haemoglobin A2
Hb F: foetal haemoglobin
MALDI-TOF MS: matrix assisted laser desorption ionization-time of flight mass spectrometry
SCD: sickle cell disease
AUC: area under the curve
ROC: receiver operating characteristic
RF: random forest
LR: logistic regression
SVM: support vector machine
KNN: K-nearest neighbour
DT: decision tree
NB: naive Bayes
Adaboost: adaptive boosting
ANN: artificial Neural Network.
