# Supplementary material for: A MALDI-TOF mass spectrometry-based haemoglobin chain quantification method for rapid screen of thalassaemia
Source: Ann Med. 2022 Jan 31;54(1):293–301. doi: 10.1080/07853890.2022.2028002 (PMC8812805; doi:10.1080/07853890.2022.2028002)
Supplement: Supplemental Material [file IANN_A_2028002_SM0891.zip › Supplemental files/Supplementary Figures.docx]

**Figure legends**

**Figure S1. The representative MALDI-TOF mass spectra of samples from thalassemia patients and control participants.** (A) The mass spectra of 1+ charged α-globin, β-globin, γ-globin and internal standard peaks. (B) The mass spectra of 2+ charged α-globin, β-globin, γ-globin and internal standard (myoglobin) peaks.

**Figure S2. Violin plot of the relative level of 10 features in thalassemia patients and controls.** Outliers are not shown for clarity.

**Figure S3. PCA analysis using the 10 features in cohort 1.**

**Figure S4. The AUC value of each feature for distinguishing α- or β-thalassemia patients in cohort 1.**

**Figure S1**


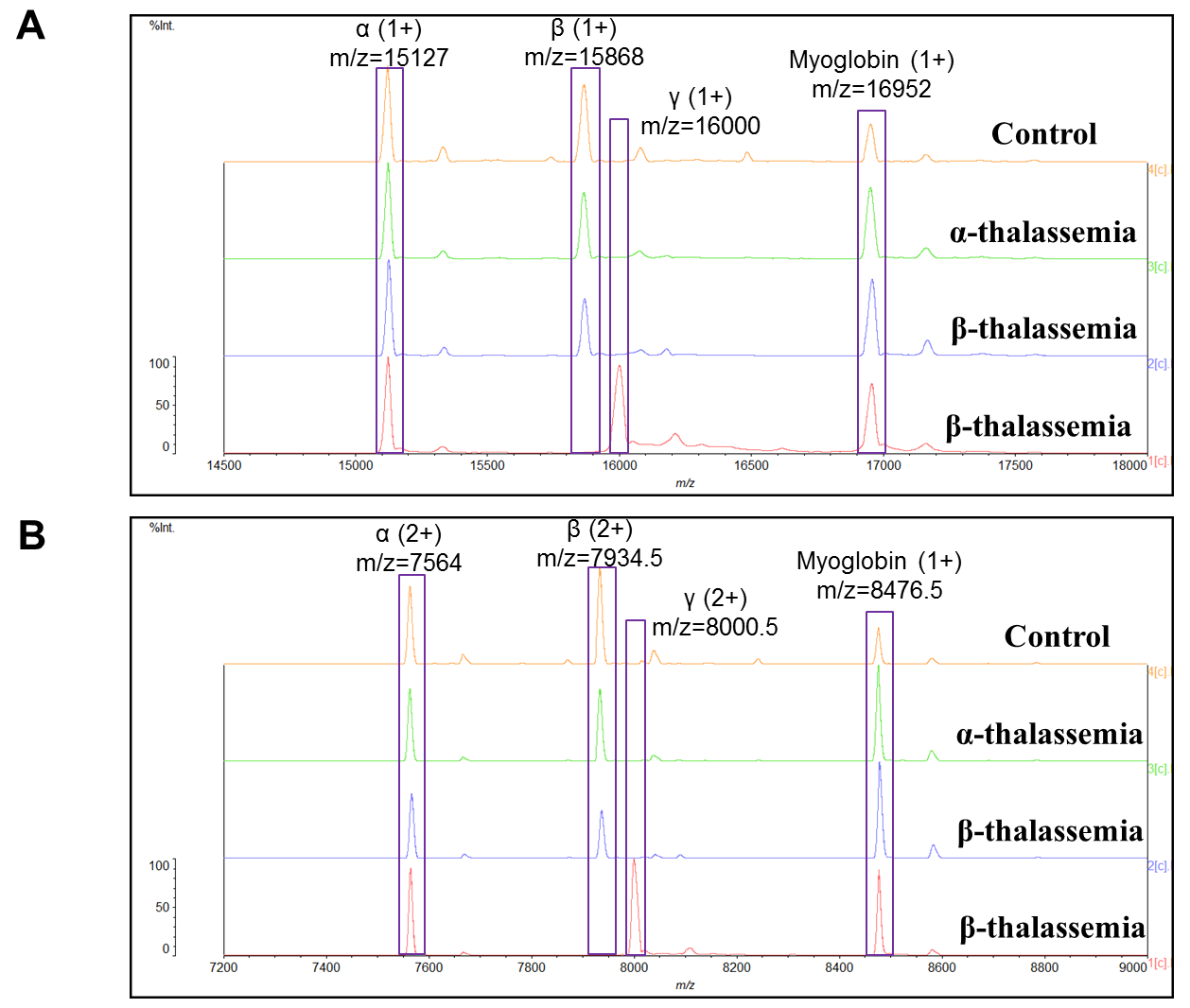


**Figure S2**


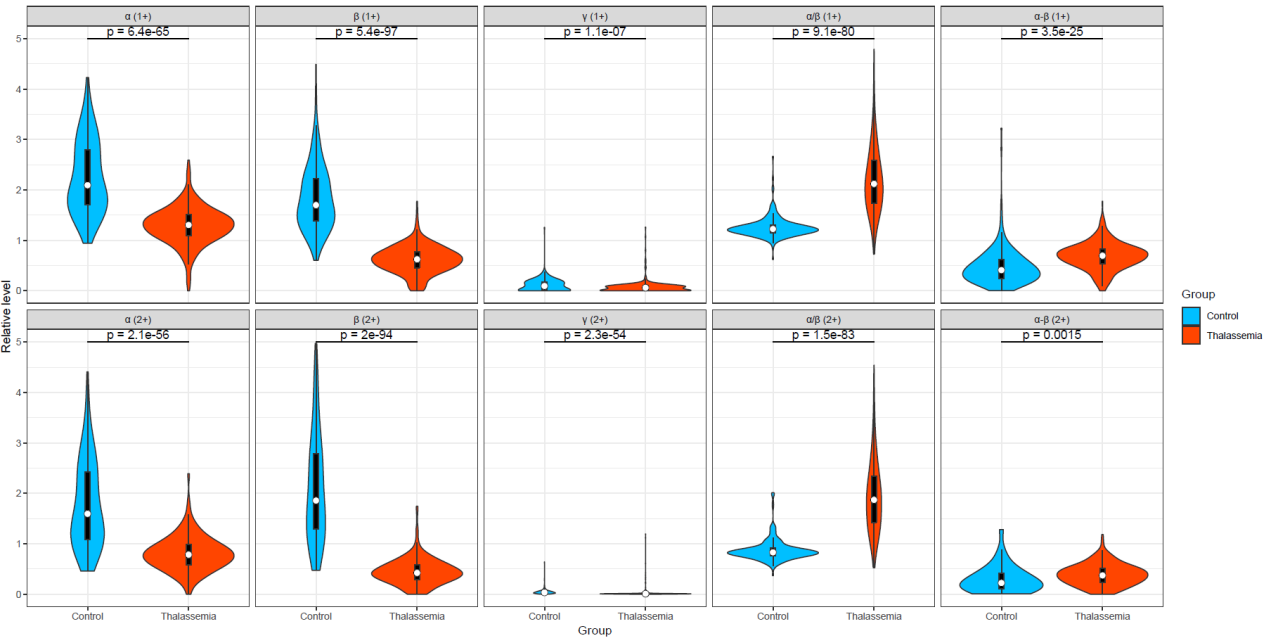


**Figure S3**


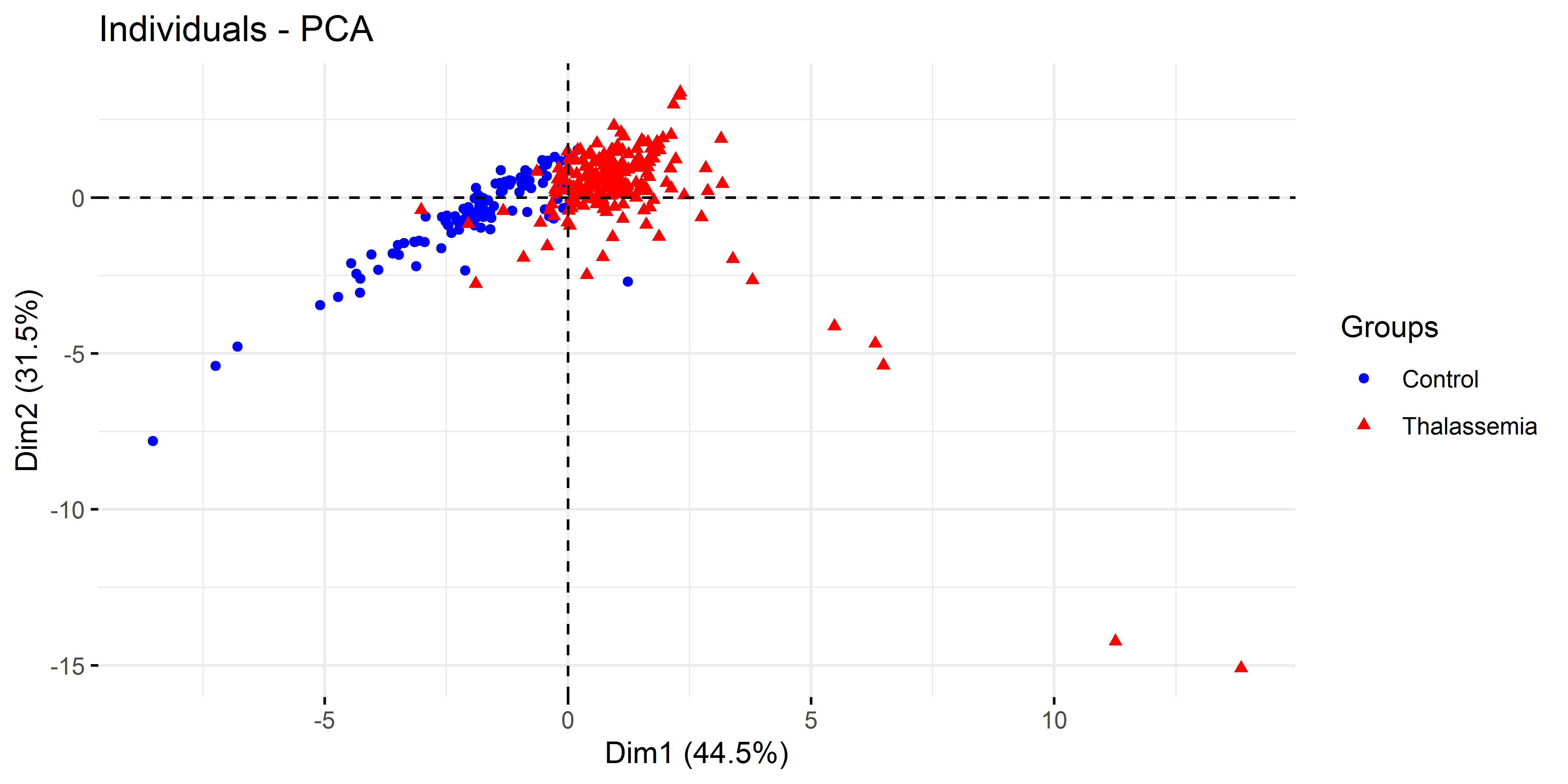


**Figure S4**
